# System dynamics simulation of occupational health and safety management causal model based on NetLogo

**DOI:** 10.1016/j.heliyon.2023.e18752

**Published:** 2023-07-27

**Authors:** Zhonghong Cao, Junjie Zhu, Binbin Tang, Tao Chen

**Affiliations:** a School of Economics and Management, Hunan University of Science and Engineering, Yongzhou 425199, Hunan, PR China; b School of Accounting, Wuhan Qingchuan University, Wuhan 430204, Hubei, PR China; c Wuhan University of Science and Technology, Wuhan 4300081, Hubei, PR China

**Keywords:** NetLogo, Management causal model, System dynamics, SEM, Occupational health and safety

## Abstract

The occupational health and safety management factors of construction enterprises are critical influencing factors in their training management, and their causal principles are topics that warrant profound exploration. Drawing upon the conventional five factors, this study initially posited and authenticated a causal model among them, subsequently employing system dynamics on the NetLogo platform to dynamically simulate the model, and ultimately scrutinizing the interrelations and dynamic influence degree among the factors. The results show that the direct causes of management factors include human factors (weight coefficient of 0.583) and method factors (weight coefficient of 0.405), and environmental factors directly affect human factors (weight coefficient of 0.994), whereas material factors directly affect method factors (weight coefficient of 0.918). At the same time, it can be seen from the dynamic simulation results that the influence of human factors and method factors on management factors increases sharply in the nascent phase of the simulation cycle (the highest slope is .90), gradually decreases in the intermediary phase (the slope of the inflection point is .11), and is relatively stable in the final phase (the slope is less than 0.11). Three main conclusions have been drawn from this. Firstly, management factors are directly and positively affected by human factors and method factors respectively. Secondly, the interplay between diverse factors evinces a confluence of periodicity and exponential attributes. Thirdly, in each management cycle (set at 381 steps), the main focus is on controlling the causal factors in the early stages of management, with pivotal control points in steps 25 and 100, and the principal management factors comprising the management organization, operating procedures, and protective measures.

## 1 Introduction

### 1.1 Background

In the management of enterprises, occupational health and safety (OHS) training management is an important part of management [1,2], while there are many factors affecting the OHS training managers at all levels of construction enterprises [3]. Given that the administration of OHS training is progressively being implemented in construction-related matters, it assumes considerable significance for the health and safety of construction workers [[4], [5], [6]]. Physical diseases and occupational health service activities of construction workers [7], psychosocial working environment, and mental health of construction workers constitute the most basic factors affecting OHS [8]. With construction enterprises entering the global competitive arena, the health and safety hazards faced by foreign site workers have escalated markedly [9,10]. Consequently, the occupational hazards in construction, particularly the OHS injury risks of construction site workers, have engendered the attention of numerous construction enterprises [11,12]. The unsafe behavior of construction workers is the main cause of injuries in accidents [13], and the establishment of an OHS Management System (OHS-MS) can provide institutional guarantees for proactive safety [14]. Augmenting OHS training could elevate the safety management standards of Chinese construction workers, thereby fulfilling the objective of diminishing fatalities in construction accidents [15], and it has also emerged as a focal point of risk management in construction enterprises [16]. Therefore, investigating various factors affecting the management of OHS training and the causal relationship between them can furnish a novel approach for enhancing the formulation of strategies for OHS training management.

### 1.2 Literature review

This study first proposed an OHS management causal model and used a structural equation model for empirical analysis to verify the validity and reliability of the ultimate model [17,18]. The classical theory holds that the main cause of accidents is the hazardous conduct of people, the unsatisfactory state of facilities [19], and an unsound management system. Recent studies have also prioritized mental health factors, especially suicide prevention among construction workers [[20], [21], [22]]. Risk perception varies according to diverse cultural backgrounds and is considerably affected by race, which impacts the working environment of laborers [23]. Concerning physiological factors, a conspicuous correlation exists between the musculoskeletal ailments of construction workers and occupational health interventions [7]. Certain scholars have investigated the cases of infectious diseases in the workplace in the United States from 2006 to 2015, revealing that laborers operating in particular settings and tasks face heightened vulnerability to infectious ailments [24,25]. The incorporation of occupational risk factors and regulation of occupational exposure can impede the propagation of diseases in the workplace and safeguard the health of workers [26]. According to the statistics and analysis of the prevalence and injury rate of occupational injuries among construction workers, gender, education level, safety training, personal protective equipment, and other factors evince significant and static correlation with management factors [27]. Therefore, the current study could advance hypothesis H1: Human factors exert a direct and positive impact on management factors.

In the safety construction plan of construction sites, scholars have adopted the classification and prediction method of occupational health, and the results show that the working environment conditions have a significant impact on workers' health [28]. Investigations have demonstrated that in “high-risk” professions, some practitioners have risk preference characteristics and tend to pursue high returns in high-risk situations [29]. Moreover, industrial accidents often occur during working hours and posts. The cases with a high injury rate for construction workers are mostly cuts, most of which are night shift injuries [30,31]. The combination of legislation, publicity, and safety education can abet in curtailing the incidence of injuries [32]. Therefore, reasonable construction schemes and conditions are important factors to enhance the management level. Consequently, this study led to hypothesis H2: Method factors have a positive and direct impact on management factors.

The main influencing factors in terms of equipment and materials include economic investment and savings in construction costs [33,34]. Inquiries and research have additionally discerned that inadequate supply and deficient management attributable to material factors will engender novel hazards at the construction site, underscoring the importance of economic investment in ensuring material factors [35]. Therefore, OHS cost control for construction projects [36] is an important constituent of material factors [37,38]. The factors affecting the safety methods of contractors include an incomplete OHS management system, a lack of training and knowledge in safety management, and the absence of SSS, SHO, or safety managers [39]. Moreover, the inadequacy of OHS system documents is also a detrimental factor for methodological factors [40]. In addition, the formulation of OHS performance indicators and performance measurement in construction methods are also important factors affecting OHS management [5,41,42]. Therefore, this study has instigated hypothesis H3: The factor of material has a direct and positive effect on the factor of the method.

In terms of environmental and climatic factors, the impact of climate change on outdoor workers and their safety is of paramount importance [43,44]. The relationship between night work and all-cause mortality of the general working population is significant [45]. In particular, the World Health Organization (WHO) and the International Labor Organization (ILO) are developing a joint methodology to estimate the national and global burden of work-related diseases and injuries (WHO/ILO joint methodology) [[46], [47], [48], [49]], with particular attention to the management and regulation of safe climate factors [50,51]. Some studies believe that construction noises also have a great impact on workers' health [52]. Therefore, this study led to hypothesis H4: Environmental factors directly affect human factors.

NetLogo is a programmable modeling environment utilized for simulating natural and social phenomena. It was launched by Uri Wilensky in 1999 and has been continuously developed by the Center for Connected Learning and Computer-Based Modeling, Northwestern University, Evanston, IL (http://ccl.northwestern.edu/netlogo/). Since the relationship between various factors of enterprise management is a complex system [53], how to explore the influence relationship and influence degree of subfactors between and within factors is one of the topics of enterprise management worth studying [[54], [55], [56]]. Although enterprise management is a complex system, it can be viewed at a high level as a group of inventory, flow, and constants, making system dynamics simulation an apt tool for modeling. In the preliminary analysis, although some internal analysis elements may be lost when using the SD model, future research can expand the modeling in greater detail on this basis. NetLogo-based system dynamics modeling can not only explore the action relationship between individual factors [[57], [58], [59]] but also demonstrate the dynamic process through curves [[60], [61], [62]]. Building upon the above management causal model, this study further adopted system dynamics modeling and simulation methods [25,31,63], to explore the dynamic influence relationship between management cause factors and internal subfactors. By virtue of the great familiarity the research team had with NetLogo, it took less effort to model with NetLogo than common SD modeling platforms which may be overkill. Therefore, this research utilized the Netlogo platform for modeling applications.

### 1.3 Research objectives and outlook

The main purpose of this study is to first construct an OHS management causal model and reveal the causal relationships between various factors that affect management factors, and then analyze the key subfactors. Secondly, the Dynamic simulation method is further employed to display the cyclical change characteristics of the main factors, providing new means and approaches for the management of OHS in construction enterprises. Therefore, this study holds certain research value.

In the following section, this study first proposes an OHS management causal hypothesis model in the methods section. Subsequently, SEM methods are used for model validation and optimization. Based on this, NetLogo system dynamics simulation is performed. Finally, the simulation results are explicated, conclusions are drawn, and practical suggestions are proposed.

## 2 Methods

### 2.1 Management causal hypothesis model

**Fig. 1:**
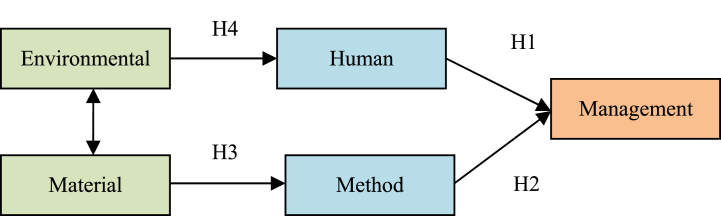
OHS management causal model.

Drawing upon previous studies, this study proposed a hypothetical model (Fig. 1). The target factor is the management factor. The source factors are the human, method, material, and environmental factors, the first two of which are directly influencing factors, and the last two are the origin factors. The four hypotheses are formulated as follows. Human factors have a positive and direct impact on management factors (H1). Method factors have a positive and direct impact on management factors (H2). The factor of material has a positive and direct effect on the factor of the method (H3). Environmental factors directly affect human factors (H4). This study first verified the hypothesis model and subsequently conducted an empirical analysis.

### 2.2 Variable design of management causal model

In this paper, codes were designed for the sub-variables of five types of factors (refer to Table 1) to be used in conjunction with the OHS management causal model (see Fig. 1), aiming at various influencing factors of OHS training management of construction enterprises. Among them, there are five types of factors. ‘SEM Code’ is the code to be used by the structural equation model method, and ‘Description’ is the description of sub-variables. The sub-variables marked with gray shading in Table 1 are the optimized variable in the subsequent SEM modeling.

**Table 1:** Factors of OHS training management.

Factor	SEM Code	Description	Abbreviation
Human	AX01	Management consciousness	MC
	AX02	Safety consciousness	SC
	AX03	Psychological quality	PQ
	BX04	Good health	GH
	BX05	Health examination	HE
	BX06	Protective equipment	PE
	BX07	Personal protection	PP
Material	AY01	Material allocation	MA
	AY02	Material quality	MQ
	AY03	Material inspection	MI
	BY04	Own equipment	OE
	BY05	Equipment maintenance	EM
	BY06	Equipment safety	ES
	BY07	Device defect	DF
	CY08	Financing	FC
	CY09	Innovation investment	II
	CY10	Protection Investment	PS
Management	AZ01	Management organization	MO
	AZ02	Organization and command	OC
	AZ03	Security personnel	SP
	AZ04	Personnel qualification	PQC
	AZ05	Safety supervision	SS
	BZ06	System implementation	SI
	BZ07	On-site system	OS
	BZ08	Operating regulations	OR
	BZ09	Operation procedure	OP
	BZ10	Preventive measures	PM
	BZ11	Emergency rescue	ER
	AT02	Training system	TS
	AT04	Education and training	ET
Method	AU01	Scheme approval	SA
	AU02	Top ten technologies	TTT
	AU03	Technical disclosure	TD
	AU04	Technical proposal	TP
	AU05	Process use	PU
	AT03	Process training	PT
Environmental	AS01	Site Layout	SL
	AS02	Climatic conditions	CC
	AS03	Geological conditions	GC
	AS04	Noise measures	NM
	BS05	Policy environment	PE
	BS06	Economic development	ED
	BS07	Surrounding environment	SE
	BS08	Project cooperation	PC
	AT01	Industry training	IT

### 2.3 Questionnaire survey and data collection

In order to verify the causal relationship and correlation between the factors in the hypothetical management causal model, the following questionnaire survey and analysis were carried out.

(1) Questionnaires

The survey targeted construction enterprises (including construction companies, supervision companies, construction companies, etc.), and covered five factors (human, material, method, environmental, and management) at the enterprise-level, project-level, and team levels. A total of 530 valid online questionnaires were collected. For details on the questionnaire design, refer to the datasets [GENERATED/ANALYZED] for this study, which can be found in [FigShare] [10.6084/m9.figshare.14431262] and Appendix. The data analysis tools used were IBM SPSS Statistics 23 for statistical analysis and AMOS Graphics 23 for structural equation analysis (SEM). The collected data can also be found in the datasets [GENERATED/ANALYZED], which can be accessed through [FigShare] [10.6084/m9.figshare.14431271].

(2) Data Source Analysis
  1) Source Channel Analysis

It can be seen from Table 2 that the questionnaire responses were obtained from three sources: WeChat, mobile submission, and link, of which WeChat accounts for the largest proportion, 71.70%.

2) Start and End Time Analysis

**Table 2:** Analysis of sources and channels of the questionnaire.

Source channel	Number of questionnaires	Percentage
WeChat	380	71.70%
Mobile	148	27.92%
link	2	0.38%
Total	530	100%

Table 3 indicates that most of the questionnaires, 509 out of 530, were collected in three days from April 25 to 27, 2020, accounting for 96.04% of the total. The recovery time of all questionnaires is a duration of two months.

(3) Indicator Description

**Table 3:** Analysis of the starting and ending time of the questionnaire.

Time	Number of questionnaires	Percentage
2020/4/25	160	30.19%
2020/4/26	161	30.38%
2020/4/27	188	35.47%
2020/4/28	12	2.26%
2020/4/29	2	0.38%
2020/4/30	2	0.38%
2020/5/1	1	0.19%
2020/5/9	1	0.19%
2020/5/24	1	0.19%
2020/5/27	1	0.19%
2020/6/22	1	0.19%
Total	530	100.00%

From the demographic description table (see Table 4), it can be seen that the number of valid questionnaires is 530. The minimum and maximum values for items 1 to 7 were 1 and 6, respectively, with an average value between 0 and 3.22 and a standard deviation between 0 and 2. The narrow deviation range indicates that the data is relatively concentrated and meets the requirements of data statistics.

**Table 4:** The demographic descriptive statistics table.

	N	Minimum	Maximum	Mean	Std. Deviation
1. What's your age?	530	1	4	2.19	1.001
2. What is your educational background?	530	1	4	2.06	.313
3. What type of work unit do you work in?	530	1	6	2.00	1.753
4. What's your position?	530	1	3	1.88	.600
5. What is your professional title?	530	1	4	3.22	.707
6. How long have you worked in the construction enterprise?	530	1	4	2.95	1.175
7. Which of the following management system certification has your company passed? (ISO9000 quality management system)	530	0	1	.72	.448
7. Which of the following management system certification has your company passed? (ISO14000 environmental management system)	530	0	1	.44	.496
7. Which of the following management system certification has your company passed? (OHSAS18000 occupational health and safety management system)	530	0	1	.43	.496
7. Which of the following management system certification has your company passed? (Passed other management system certification)	530	0	1	.22	.413
7. Which of the following management system certification has your company passed? (Failed to pass any management system certification)	530	0	1	.14	.347
Valid N (listwise)	530				

In addition, the frequency analysis shows that the age of the respondents to the questionnaire is relatively balanced (30–50 years old). Among the academic qualifications, university accounted for the highest percentage, 91.0% (including undergraduate and junior college). Among the working units (construction unit, supervision unit, construction unit, university, government, and others), the construction unit accounted for 69.8%. In terms of professional titles, 24.5% of the respondents were company-level managers, 62.6% were project-level managers, and 12.8% were team-level managers. In addition, the intermediate professional title was the most common, accounting for 63.7% of the responses. The majority of respondents (53.6%) had worked in construction enterprises for 6–9 years. Notably, only 43.3% of the enterprises had passed the OHS management system, indicating a need for strengthened promotion of the occupational health management system in construction enterprises.

## 3 Result

### 3.1 Statistical description and SEM model evaluation

Next, items 8 to 52 of the questionnaire will be statistically described and analyzed according to the five major categories of factors.

(1) Validity analysis (KMO and Bartlett test)

1) *Content Validity Analysis.* Content validity, also known as logical validity, refers to the appropriateness of an item's sampling of the content or range of behavior to be measured, that is, the appropriateness and consistency of the measured content. Part of the questionnaire in this study drew lessons from the scales that have been used many times and had good results in domestic and international research, while the rest were self-developed, whose scale was summarized through the example of OHS training in construction enterprises. Before the final questionnaire was determined, some contents were modified and improved through discussion with respondents and relevant experts. Subsequent analysis can ensure the good content validity of the questionnaire in this study (see Table 2.).

2) *Structural Validity Analysis.* Structural validity refers to the degree to which a test actually measures the theoretical structure and traits to be measured, and refers to the consistency between the experiment and the theory, that is, whether the experiment truly measures the hypothesis (construction) of the theory. Due to the large number of variables in this model, it is necessary to conduct an exploratory factor analysis to group the variables in the model. In this study, the principal component analysis method was used to group the influencing factors with SPSS 23.0 software. Exploratory factor analysis was performed on each factor, which was forcibly divided into several principal component factors, and analyzed by maximizing variance orthogonal rotation. Before factor analysis, the KMO (Kaiser-Meyer-Olkin) test and the Bartlett sphere test were conducted with SPSS 23.0 software to assess whether the variables were suitable for factor analysis. The test results in this study showed that (see Table 5.) the KMO sample test value of each scale was greater than 0.7, and the Bartlett sphere test value was less than 0.001. Therefore, factor analysis can be carried out on the collected data [64].

**Table 5:** KMO and Bartlett's test.

Kaiser-Meyer-Olkin Measure of Sampling Adequacy.		.979
Bartlett's Test of Sphericity	Approx. Chi-Square	26832.231
	df	990
	Sig.	.000

Table 5 shows the results of the exploratory factor analysis on the questionnaire items. Each measurement item was well-distributed on several potential common factors, with a factor load greater than 0.5, while the factor load on other potential variables was less than 0.5. This indicates good convergence and differential validity of the questionnaire data. When the independent variables with a factor load less than 0.5 were removed, the results of exploratory factor analysis are shown in table. It can be seen that each measurement item in the questionnaire can be well distributed on several potential common factors, that the factor load of each measurement item is > 0.5, and that the factor load on other potential variables is < 0.5, further confirming the good convergence and differential validity of the questionnaire data [65].

(2) Reliability Analysis

Reliability refers to the degree of consistency in the results obtained by repeated measurements of the same object using the same method. After exploratory factor analysis, this study employed SPSS 23.0 statistical analysis software and Cronbach's α Coefficient to assess the internal consistency of the questionnaire [66]. The inspection results are shown in the table. The coefficients are greater than 0.6 (see Table 6.), which proves that the reliability of factors and variables in the questionnaire is high [64].

**Table 6:** Reliability statistics.

Cronbach's Alpha	Cronbach's Alpha Based on Standardized Items	N of Items
.985	.985	45

Based on the results of the above hypothetical model, the OHS management causal model was found to be effective, reliable, and of strong practical significance.

The results of the above questionnaire were then used to construct, verify, and optimize the SEM model. The optimized SEM results were imported into the SD platform of NetLogo for further analysis and display.

### 3.2 SEM optimization results

**Fig. 2:**
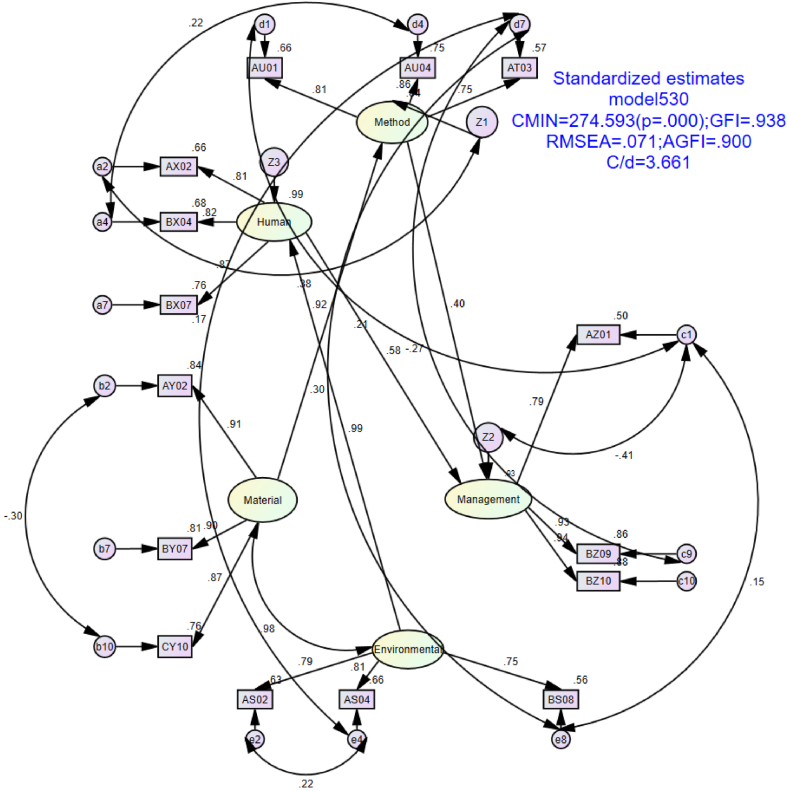
SEM measurement chart.

The SEM modeling was adopted and the data set was input into the model. The simplified measurement model can be obtained through variable and error adjustment, as shown in Fig. 2. The model optimization mainly involved the following steps. First, input all data into the model, and retain the three sub-variables with the largest square multiple correlations: estimate value among the respective sub-variables of the five types of factors (Usually, a value greater than 0.7 is selected). Then, optimize the modification indexes: M.I. value and simplify the SEM structure diagram as much as possible. Finally, if the standardized regression weights are within a reasonable range, the model optimization is considered completed.

Specifically, the variables in the model were designed and identified by combining expert interviews and literature analysis. The sub-variables of the five factors, including human factor, material factor, management factor, technical factor, and environmental factor, could be derived from the variable design items of the questionnaire (see Table 1). For example, the sub-variables of the Human factor after SEM optimization are AX02, BX04, and BX07. The other sub-variables with gray shading in Table 1 represent the input data after modeling optimization. In addition, AX01, AX03, AX05, and AX06 were not included in the sub variables of human factors in the model after model measurement and optimization, combined with expert interview opinions and the above literature review.

### 3.3 Analysis of SEM verification results

It is assumed that the model needs to be analyzed by Standardized Regression Weights and Model Fit Summary to verify the fitting results.

#### 3.3.1 Standardized regression weights

It can be seen in Table 7 that the estimated value is between - 1 and 1, which meets the requirements of standardized value. Wherein, the weight coefficient of Material to Method is 0.918, the weight coefficient of Environmental to Human is 0.994, the weight coefficient of Method to Management is 0.405, and the weight coefficient of Human to Management is 0.583, the four weights are all between - 1 and 1, and all are greater than 0.

**Table 7:** Standardized Regression Weights: (Group number 1 - Management model530).

			Estimate
Method	<---	Material	.918
Human	<---	Environmental	.994
Management	<---	Method	.405
Management	<---	Human	.583

#### 3.3.2 Model Fit Summary

It can be seen in Table 8 that the CMIN fitting value is small. But the p-value is rejected because the sample N value is large (530) [67]. The GFI value (. 938) is greater than the standard value (. 90), and the RMSEA value (. 071) is less than 0.08, indicating an acceptable fit. The AGFI value also meets the requirements of 0.90 standard value. The C/d value of 3.661 is within the range of a standard value of 5 [64]. Therefore, it can be assumed that the model fitting result is good.

**Table 8:** Model fit summary.

Parameter	Fitting value	Standard value	N
CMIN	274.593(P = 0.000)	The smaller the fitting value, the better (P > 0.05, the larger the sample size, the easier to be rejected)	530
GFI	.938	.90	
RMSEA	.071	.05（. 08 Acceptable）	
AGFI	.900	.90	
C/d	3.661	5	

Combining Table 7, Table 8, the following conclusions can be drawn. The causal weight coefficient between humans and management is 0.583, supporting the truth of hypothesis H1. The causal weight coefficient between the method and management is 0.405, indicating the support of hypothesis H2. The causal weight coefficient between material and human is 0.918, supporting hypothesis H3. The causal weight coefficient between the environment and humans is 0.994, validating hypothesis H4. As can be seen from Fig. 2, the correlation coefficient between material and environmental is 0.983(see Fig. 2.), indicating a strong correlation between these two exogenous latent variables.

The results of the questionnaire were used to verify the hypothesis model via SEM, and the weights of the factors obtained from the SEM were imported to the SD platform of NetLogo for application.

### 3.4 NetLogo system dynamics simulation

#### 3.4.1 System dynamics modeling

**Fig. 3:**
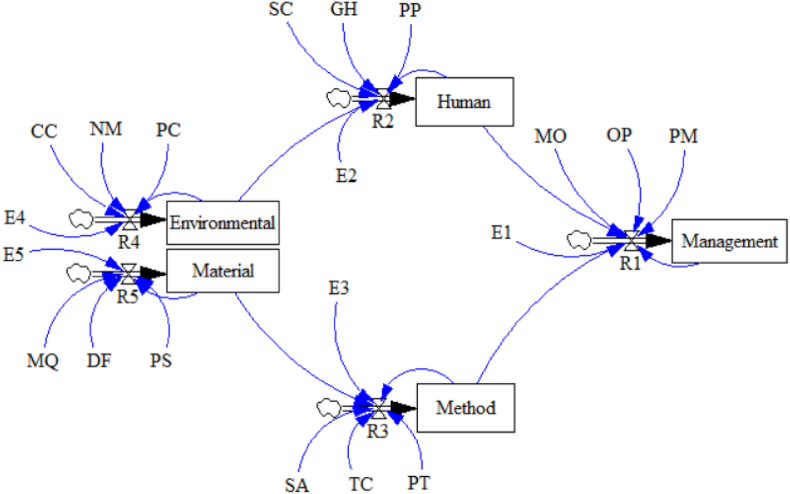
SD model.

Firstly, the optimized model in Fig. 2 was modeled on the system dynamics of the NetLogo platform. The SD flow diagram is shown in Fig. 3. It can be seen that the five factors (called stocks) have three input variables (called flows). For example, the input flow variables of stock management include MO, OP, and PM. In the figure, E1 ∼ E5 represents the expected variable values of the stocks and R1 ∼ R5 represents the rate variables. The causality between the five stocks is the causality verified by the SEM model, and the path weight value of the SEM model is directly introduced into the SD flow diagram (for example, the causality coefficient from human to management is 0.583). For the comprehensive program code text, please refer to the supporting material data set: https://doi.org/10.6084/m9.figshare.19807879.v1.

A sample code is as follows:

*;;Report value of flow*
*to-report R1*
report (ln((E1 - Management)/(MO + OP + PM + 0.583 * Human + 0.405 * Method))
) * dt
*end*

#### 3.4.2 NetLogo design and operation simulation

**Fig. 4:**
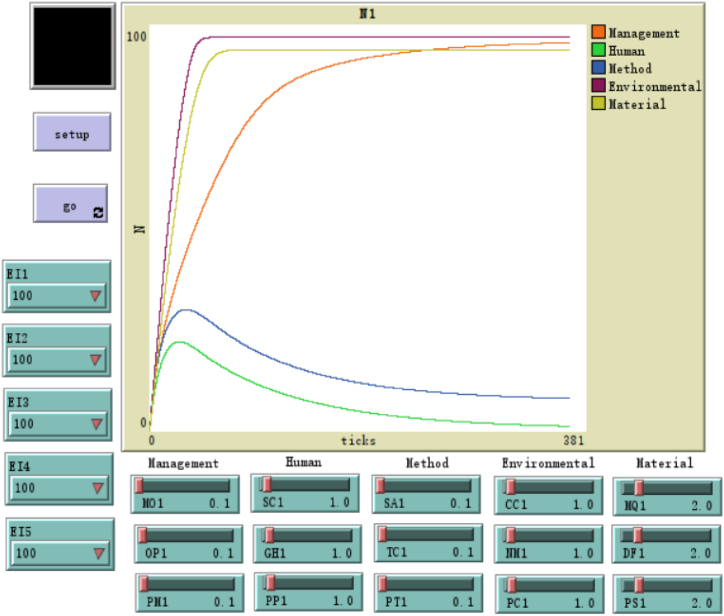
NetLogo design.

The SD flow chart simulation operation result graph can be seen in Fig. 4, in which the initial value of the expected variable E1 is expressed as EI1, the design value is 100, and the same applies to E2 ∼ E5. The initial value of the input flow variable MO of stock management is expressed as MO1, and the design value is 0.1. Other initial values OP1 and PM1 are the same. Through the simulation test run, to clearly distinguish the output curve, the values of the three input flow variables of other stock variables human, method, environmental, and material are 1.0, 0.1, 1.0, and 2.0 respectively.

Among the five curves in the figure, human and method curves show a life cycle parabola. They rise sharply within 25 ticks (the highest slope is .90), decline slowly between 25 and 100 ticks (the slope of the inflection point is .11), and are relatively stable above 100 ticks (the slope is less than 0.11). The environmental and material curves show a rising exponential curve, which rises sharply within 25 steps of ticks (with a maximum slope of 1.94) and approaches the expected value of 100 steadily above 25 steps. The management curve also presents an upward exponential curve, rising rapidly within 100 steps (with a maximum slope of .78). But above 100 steps, it is also stable and close to the expected value of 100.

Combined with Fig. 1, Fig. 4, the direct influencing factors of management factors are the human and method ones. In the early stage of the simulation cycle, human and method factors have the greatest cumulative impact on management factors. This impact is moderate in the middle stage, while is small in the later stage. Therefore, human and method factors have the greatest impact on enterprise management in the early stage of the management evaluation cycle, that is, it is necessary to strengthen the early management of human and method factors, to obtain better management effects. In addition, environmental and material factors have a positive and direct impact on human and method factors respectively, and also have a great impact on human and method factors in the early stage of the simulation cycle. To sum up, the management of various factors in the early stage of the simulation cycle is particularly important.

Through the hypothesis model and verification, it can be seen that the input variables of management factors are MO, OP, and PM. And through the simulation operation, it can be seen that adjusting the values of these three variables will bring significant fluctuations to the management curve, so it is necessary to strengthen the control of these three input factors.

In addition, to verify the sensitivity of the system dynamics model built, the input values of the three sub-factors SC, GH, and PP of the human factor were adjusted to 1.5, which is 50% higher than the simulation value of 1.0. The verification results show that the graphical relationship and trends of the model output have not changed. Similarly, adjusting the input values of sub-factors of other Methods, Environmental and Material factors shows that the dynamic model is stable and insensitive.

## 4 Discussion

Due to the numerous influencing factors on the training and management of occupational health and safety in construction enterprises, many of which are also sources of danger, the main goal of this study is to identify the main factors that affect the level of training and management based on the relevant background of construction enterprises.

In comparison, previous research on the impact of construction enterprise management, both domestically and internationally, has mainly focused on two factors or single causal relationships. However, there has been relatively little research on the causal factors in the field of occupational health and safety training management in construction enterprises. Moreover, the exploration of the dynamic impact relationship and degree of influence between factors in the causal model is the biggest difference between this study and previous studies [1]. Therefore, this article proposes for the first time an OHS management causal model in this domain, to explore and verify the principles and processes of human, material, method, and environmental factors that affect management. Finally, the model is imported into the Netlogo platform for system dynamics modeling and operation simulation, resulting in the acquisition of the dynamic impact curve within the management cycle, which proposes novel methods for enhancing the management level of occupational health and safety training in enterprises [[68], [69], [70]].

One of the primary innovations of this study compared to the preceding literature is the proposal and establishment of an OHS management causal model and the analysis and comparison of the relationships between various sub-factors in the model. The leading sub-factors of management factors include MO, OP, and PM. The sub-factors of human factors include SC, GH, and PP. The sub-factors of method factors include SA, PT, and TP. The sub-factors of material factors include MP, DF, and PI. The sub-factors of environmental factors include CC, NM, and PC. These sub-factors are the key control objects of training management.

Furthermore, this study explored the dynamic impact of various factors. From the results of system dynamics simulation, it is evident that in a management cycle, the impact of human factors and method factors on management factors increases sharply over time in the early stage, slowly decreases in the middle stage, and relatively stabilizes in the later stage. The impact curve of the lifecycle type indicates that the influencing factors and degree of management factors need to be controlled in the early stage. In addition, environmental and material factors also have a significant impact in the early stages of the management cycle and remain stable in the later stages. However, the curve presents an exponential form, indicating that the impact trend of these two factors continues to increase. Especially the management factor curve is still an exponential curve with a cumulative increase, but it has a significant impact in the early and middle stages and is relatively stable in the later stages. The enlightenment of the Dynamic simulation results is that the factors in the OHS management causal model are particularly important in the early management and control of the management cycle, but the management in the middle cannot be ignored, and the factor control in the later period has less impact on the management factors. Therefore, the occupational health and safety training management of enterprises has the characteristics of combining periodicity and index characteristics, and managers should focus on the dynamic control process and key points of five factors in the early, middle, and late stages of management.

Therefore, the OHS management causal model proposed in this study not only shows the causality of other categories of factors affecting management factors but also reveals the key sub-factors in training management. In particular, the further dynamic simulation shows the comprehensive characteristics and methods of management, providing new methods and ways for the training management of OHS in construction enterprises and other aspects of management.

However, the shortcomings of this study mainly lie in the empirical analysis of the field of construction enterprises. In the future, attempts will be made to validate other types of enterprises and promote the application of OHS management causal models to general management theory. This is also the limitation of this study.

## 5 Conclusion

The principal findings of this study can be summarized as follows:

I. The constructed OHS management causal model is reliable, revealing the causal relationships between five types of factors, including both the direct and indirect causal factors of management factors. The direct causes of management factors consist of human and methodological factors, whereas the indirect causes include environmental and material factors. It is apparent that the direct cause necessitates key control.
II. The key sub-factors of each of the five types of factors also need to be paid attention to, especially the key sub-factors of management factors, human factors, and method factors.
III. Early management is the principal control stage throughout the entire lifecycle of management, as the degree of influence of the five types of factors increases sharply during this stage. But the management in the middle and later stages cannot be ignored.

In response to the above conclusions, this article proposes the following suggestions:

I. Enterprise managers should initially concentrate on the management of Man and Method factors in actual OHS management, and enhance the analysis and tracking of all influencing factors in the early stages of each management cycle (such as annually and monthly). Secondly, control the sub-factors of various factors as key management sub-factors. For instance, strengthening management and control of the sub-factors SC, GH, and PP of Human factors, that is, focusing on safety consciousnesses, good health, and personal protection. This method can also be applied to management applications for the key sub-factors of other factors.
II. Enterprise managers play a crucial role in the early management of each management cycle and need to augment the early management of various risk factors to achieve superior management outcomes.

## Author contribution statement

Zhonghong Cao: Conceived and designed the experiments; Analyzed and interpreted the data; Contributed reagents, materials, analysis tools or data; Wrote the paper.

Junjie Zhu: Performed the experiments.

Binbin Tang: Contributed reagents, materials, analysis tools or data.

Tao Chen: Contributed reagents, materials.

## Data availability statement

Data associated with this study has been deposited at The datasets [GENERATED/ANALYZED] for this study can be found in [FigShare] [10.6084/m9.figshare.14431262]

The datasets [GENERATED/ANALYZED] for this study can be found in [FigShare] [10.6084/m9.figshare.14431271]

The datasets [GENERATED/ANALYZED] for this study can be found in [FigShare] [10.6084/m9.figshare.19807879].

## Funding

This study was funded by the 2023 Hunan Social Science Achievement Evaluation Committee Project (XSP2023GLC113), the Research Achievements in the Stage of Education Science Planning in Hunan Province, Research on the Path of Occupational Health and Safety Education for College Students under the Overall National Security Concept (Project Approval Number: XJK23BGD042), and the University level project of Hunan University of Science and Engineering (2022028).

## Ethics statement

The studies involving participants were reviewed and approved by the Research Ethics Committee of Hunan University of Science and Engineering. The participants provided written informed consent to participate in this study.

## Declaration of competing interest

The authors declare that they have no known competing financial interests or personal relationships that could have appeared to influence the work reported in this paper.
