# Supplementary material for: System dynamics simulation of occupational health and safety management causal model based on NetLogo
Source: Heliyon. 2023 Jul 27;9(8):e18752. doi: 10.1016/j.heliyon.2023.e18752 (PMC10404758; doi:10.1016/j.heliyon.2023.e18752)
Supplement: Multimedia component 1 [file mmc1.doc]

**Supplementary A**

**Questionnaire of occupational health and safety training in construction enterprises**

Dear Sir/ Madam,

Hello! First of all, thank you very much for your time! The purpose of this survey is to understand the operation status of occupational health and safety training in construction enterprises. There is no right or wrong answer to the question, only reflecting personal intention. This survey is handled anonymously. The survey data are only used for academic research and will not affect your work. Thank you for your support and cooperation.

April 25, 2020

**A.essential information**

**1. What's your age? [multiple choice question]**

□Less than 30 years old □30-39 years old □40-49 years old □50 years old and above

**2. What is your educational background? [multiple choice question]**

□High school and below □University (undergraduate, junior college) □master □doctor

**3. What type of work unit do you work in? [multiple choice question]**

□Construction unit □supervision unit □Owner unit □University Research Institute □Government □Others

**4. What's your position? [multiple choice question]**

□Company level (or head office) manager □project department (or branch) manager □team (or worker)

**5. What is your professional title? [multiple choice question]**

□Senior high □secondary high □intermediate □primary and below

**6. How long have you worked in the construction enterprise? [multiple choice question]**

□Less than 2 years □2-5 years □6-9 years □10 years and above

**7. Which of the following management system certification has your company passed? [multiple choice questions]**

□ISO9000 quality management system □ISO14000 environmental management system □OHSAS18000 occupational health and safety management system □Passed other management system certification □Failed to pass any management system certification

**B. Questionnaire content**

According to your knowledge and experience, please judge the different factors influencing occupational health and safety training in the following items from different levels. 1-5 stands for different degrees of influence, among which 1 stands for the smallest degree of influence and 5 stands for the largest degree of influence.

**Influence degree**

**Number Item Very small, relatively small, average, relatively large, very large**

**1) Company management level**

**GA. Human factors**

8. the management awareness of occupational health and safety of the company's managers is insufficient [scale] 12345

9. the number of professional (practicing) qualifications of the company's practitioners is insufficient [scale] 12345

10. the company management system is not implemented effectively [scale] 12345

**GB. Material Factors**

11. the company has insufficient ability to allocate materials and materials [scale] 12345

12. the number of equipment and machinery owned by the company is too small [scale] 12345

13. limited company funds and financing means [scale] 12345

**GC. Environmental factors**

14. the policy and environment of the company's location are unfavorable [scale] 12345

15. underdeveloped economic development of the company's location[scale] 12345

16. the competent department of the industry where the company is located does not attach importance to the training of occupational health and safety management [scale]1 2 3 4 5

**GD. Method factors**

17. the company has not strictly approved the special scheme with high risk [scale] 12345

18. the company is not able to promote the ten new technologies of construction engineering [scale] 12345

19. the company has insufficient human and financial investment in technological innovation [scale] 12345

**GE. Management factors**

20. the unreasonable setting of safety management organization of the company [scale] 12345

21. the company has insufficient supervision and management on safety [scale] 12345

22. the company's occupational health and safety training system is incomplete [scale] 12345

**2) Project management level**

**XA. Human factors**

23. the safety awareness of the site manager is insufficient [scale] 12345

24. improper organization and command of site management [scale] 12345

25. the implementation of the site management system is not strong [scale] 12345

**XB. Material Factors**

26. the control of material entry inspection is not strict [scale] 12345

27. equipment maintenance is missing in regular maintenance [scale] 12345

28. insufficient supply of safety protection articles [scale] 12345

**XC. Environmental factors**

29. the construction site layout is not standardized [scale] 12345

30. the construction environment around the project site is poor [scale] 12345

31. the cooperation strength of the project partner is not high [scale] 12345

**XD. Method factors**

32. technical disclosure is not careful [scale] 12345

33. the construction technical scheme is missing for the projects with a strong specialty [scale] 12345

34. the new technology and new process training of the project department is missing [scale] 12345

**XE. Management factors**

35. the safety production management personnel are not in place [scale] 12345

36. the emergency rescue management on the construction site is not in place [scale] 12345

37. the rules and regulations of safe operation on site are not perfect [scale] 12345

**3) Team level**

**BA. Human factors**

38. the psychological quality of construction personnel is insufficient [scale] 12345

39. poor health of construction personnel [scale] 12345

40. the construction personnel did not wear personal protective articles correctly [scale] 12345

**BB. Material Factors**

41. safety hazards of site materials [scale] 12345

42. defects in the quality of field mechanical equipment [scale] 12345

43. defective field protective articles or devices [scale] 12345

**BC. Environmental factors**

44. severe cold, high temperature, thunderstorms, and other bad climatic conditions [scale] 12345

45. poor geological conditions such as hydrology and geomorphology [scale] 12345

46. improper measures for dust and noise prevention [scale] 12345

**BD. Method factors**

47. improper operation procedure [scale] 12345

48. accident prevention measures error [scale] 12345

49. improper use of new equipment and new process [scale] 12345

**BE. Management factors**

50. lack of vocational education and training for construction personnel [scale] 12345

51. the construction personnel lack pre-job health examination [scale] 12345

52. insufficient investment in safety protection measures on construction site [scale] 12345

53. your suggestions for this questionnaire are as follows: .

This questionnaire is over. Once again, I would like to express my sincere thanks to you for your support. I wish you a good job and a happy life.
