# Supplementary material for: System dynamics simulation of occupational health and safety management causal model based on NetLogo
Source: Heliyon. 2023 Jul 27;9(8):e18752. doi: 10.1016/j.heliyon.2023.e18752 (PMC10404758; doi:10.1016/j.heliyon.2023.e18752)
Supplement: Multimedia component 2 [file mmc2.doc]

**Supplementary B**

;; System dynamics model globals

globals [

;; stock values

Environment

Human

Management

Material

Method

;; size of each step, see SYSTEM-DYNAMICS-GO

dt

]

;; Initializes the system dynamics model.

;; Call this in your model's SETUP procedure.

to system-dynamics-setup

reset-ticks

set dt 0.001

;; initialize stock values

set Environment 0

set Human 0

set Management 0

set Material 0

set Method 0

end

;; Step through the system dynamics model by performing next iteration of Euler's method.

;; Call this in your model's GO procedure.

to system-dynamics-go

;; compute variable and flow values once per step

let local-MO MO

let local-OP OP

let local-PM PM

let local-SC SC

let local-GH GH

let local-PP PP

let local-SA SA

let local-TC TC

let local-PT PT

let local-CC CC

let local-NM NM

let local-PC PC

let local-MQ MQ

let local-DF DF

let local-E1 E1

let local-E2 E2

let local-E4 E4

let local-E5 E5

let local-E3 E3

let local-PS PS

let local-R1 R1

let local-R2 R2

let local-R3 R3

let local-R4 R4

let local-R5 R5

;; update stock values

;; use temporary variables so order of computation doesn't affect result.

let new-Environment ( Environment + local-R4 )

let new-Human ( Human + local-R2 )

let new-Management ( Management + local-R1 )

let new-Material ( Material + local-R5 )

let new-Method ( Method + local-R3 )

set Environment new-Environment

set Human new-Human

set Management new-Management

set Material new-Material

set Method new-Method

tick-advance dt

end

;; Report value of flow

to-report R1

report ( ln((E1 - Management) / (MO + OP + PM + 0.583 * Human + 0.405 * Method))

) * dt

end

;; Report value of flow

to-report R2

report ( ln((E2 - Human) / (SC + GH + PP + 0.994 * Environment))

) * dt

end

;; Report value of flow

to-report R3

report ( ln((E3 - Method) / (SA + TC + PT + 0.981 * Material))

) * dt

end

;; Report value of flow

to-report R4

report ( ln((E4 - Environment) / (CC + NM + PC))

) * dt

end

;; Report value of flow

to-report R5

report ( ln((E5 - Material) / (MQ + DF + PS))

) * dt

end

;; Report value of variable

to-report MO

report MO1

end

;; Report value of variable

to-report OP

report OP1

end

;; Report value of variable

to-report PM

report PM1

end

;; Report value of variable

to-report SC

report SC1

end

;; Report value of variable

to-report GH

report GH1

end

;; Report value of variable

to-report PP

report PP1

end

;; Report value of variable

to-report SA

report SA1

end

;; Report value of variable

to-report TC

report TC1

end

;; Report value of variable

to-report PT

report PT1

end

;; Report value of variable

to-report CC

report CC1

end

;; Report value of variable

to-report NM

report NM1

end

;; Report value of variable

to-report PC

report PC1

end

;; Report value of variable

to-report MQ

report MQ1

end

;; Report value of variable

to-report DF

report DF1

end

;; Report value of variable

to-report E1

report EI1

end

;; Report value of variable

to-report E2

report EI2

end

;; Report value of variable

to-report E4

report EI4

end

;; Report value of variable

to-report E5

report EI5

end

;; Report value of variable

to-report E3

report EI3

end

;; Report value of variable

to-report PS

report PS1

end

;; Plot the current state of the system dynamics model's stocks

;; Call this procedure in your plot's update commands.

to system-dynamics-do-plot

if plot-pen-exists? "Environment" [

set-current-plot-pen "Environment"

plotxy ticks Environment

]

if plot-pen-exists? "Human" [

set-current-plot-pen "Human"

plotxy ticks Human

]

if plot-pen-exists? "Management" [

set-current-plot-pen "Management"

plotxy ticks Management

]

if plot-pen-exists? "Material" [

set-current-plot-pen "Material"

plotxy ticks Material

]

if plot-pen-exists? "Method" [

set-current-plot-pen "Method"

plotxy ticks Method

]

end
